# Molecular Phylogeny of the Acanthocephala (Class Palaeacanthocephala) with a Paraphyletic Assemblage of the Orders Polymorphida and Echinorhynchida

**DOI:** 10.1371/journal.pone.0028285

**Published:** 2011-12-05

**Authors:** Lisa Verweyen, Sven Klimpel, Harry W. Palm

**Affiliations:** 1 Biodiversity and Climate Research Centre (BiK-F), Medical Biodiversity and Parasitology; Senckenberg Gesellschaft für Naturforschung (SGN); Goethe-University (GO), Institute for Ecology, Evolution and Diversity, Frankfurt/Main, Germany; 2 Aquaculture and Sea-Ranching, Faculty of Agricultural and Environmental Sciences, University Rostock, Rostock, Germany; University of Western Ontario, Canada

## Abstract

Acanthocephalans are attractive candidates as model organisms for studying the ecology and co-evolutionary history of parasitic life cycles in the marine ecosystem. Adding to earlier molecular analyses of this taxon, a total of 36 acanthocephalans belonging to the classes Archiacanthocephala (3 species), Eoacanthocephala (3 species), Palaeacanthocephala (29 species), Polyacanthocephala (1 species) and Rotifera as outgroup (3 species) were analyzed by using Bayesian Inference and Maximum Likelihood analyses of nuclear 18S rDNA sequence. This data set included three re-collected and six newly collected taxa, *Bolbosoma vasculosum* from *Lepturacanthus savala*, *Filisoma rizalinum* from *Scatophagus argus*, *Rhadinorhynchus pristis* from *Gempylus serpens*, *R. lintoni* from *Selar crumenophthalmus*, *Serrasentis sagittifer* from *Johnius coitor*, and *Southwellina hispida* from *Epinephelus coioides*, representing 5 new host and 3 new locality records. The resulting trees suggest a paraphyletic arrangement of the Echinorhynchida and Polymorphida inside the Palaeacanthocephala. This questions the placement of the genera *Serrasentis* and *Gorgorhynchoides* within the Echinorhynchida and not the Polymorphida, necessitating further insights into the systematic position of these taxa based on morphology.

## Introduction

The endoparasitic phylum Acanthocephala Kohlreuther, 1771 consists of about 1,150 species, belonging to 125 genera [1] and 19 families [2]. They are characterized by an evertable proboscis as the attachment organ, sexual dimorphism, males with cement glands and an uterine bell in females. Unique is the syndermatic tegument, placing the acanthocephalans, also confirmed by molecular studies, sister to the Rotifera [3], [5]. Recent classifications distinguish the four classes Archiacanthocephala, Eoacanthocephala, Palaeacanthocephala and Polyacanthocephala [2], [6]–[10], with a majority of 62.7% of the species primarily infecting aquatic hosts [1]. Around 57% species of the Acanthocephala belong to the Palaeacanthocephala [1] with the two orders Echinorhynchida and Polymorphida. They show the highest species diversity and are the most common acanthocephalans of marine teleost fish.

Earliest molecular data of the Acanthocephala were based on a single acanthocephalan taxon used as an outgroup to estimate the phylogenetic position of the Chaetognatha amongst the Metazoa [11]. The first molecular phylogenetic analyses inside the Acanthocephala [12] confirmed the major taxonomic grouping of the traditional classifications. There, Palaeacanthocephala placed close to the Eoacanthocephala, with the Archiacanthocephala being the most basal taxon. The bird parasitic Archiacanthocephala and Eoacanthocephala (parasites of fish, amphibians and reptiles) appeared on different branches on the resulting rDNA tree [13], [14], indicating independent evolution. Furthermore, the phylogenetic analyses suggested very complex evolutionary and taxonomic relationships among the species [12]. With their relatively small number of species, a conserved two-host (arthropod–vertebrate) life cycle, and corroborated phylogenetic relationships to a free-living sister group (the Rotifera), the acanthocephalans are attractive candidates as model organisms for studying the ecology and co-evolutionary history of parasitic life cycles in marine ecosystem. However, with many genera having only a single representative, few researchers collected specimens for molecular studies. With poor representation especially of marine taxa, the phylogenetic relationships within this interesting phylum are far from getting resolved.

Most previous analyses of acanthocephalan phylogenetic relationships have been based exclusively on nuclear small subunit (SSU) ribosomal DNA (rDNA). This highly conserved region is best suited for an analysis of the upper level phylogeny. García-Valera and Nadler [4], [9] analyzed a total of 21 acanthocephalan species, including 3 Archiacanthocephala, 2 Eoacanthocephala, 15 Palaeacanthocephala and 1 Polyacanthocephala. The purpose of the present study was to add new sequence data especially of marine fish parasitic taxa, providing a better resolution inside the Palaeacanthocephala. This is a prerequisite for a better understanding of this taxon, also enabling a better taxonomic placement and morphological identification of the species within this group. Marine acanthocephalans from different sources were collected, morphologically identified, and analyzed for the nearly complete 18S rDNA. Five of these species have not been included in molecular phylogenetic analyses before (*Bolbosoma vasculosum*, *Filisoma rizalinum*, *Rhadinorhynchus prists*, *R. lintoni* and *Serrasentis sagittifer*). The available sequence data of 29 Palaeacanthocephala, 3 Eoacanthocephala, 3 Archiacanthocephala, a single Polyacanthocephala, and three from Rotifera as outgroup were analyzed by Bayesian Inference and Maximum Likelihood. Implications for the phylogeny of the marine acanthocephalans are discussed.

## Results

### Species identification and data set

**Figure 1:**
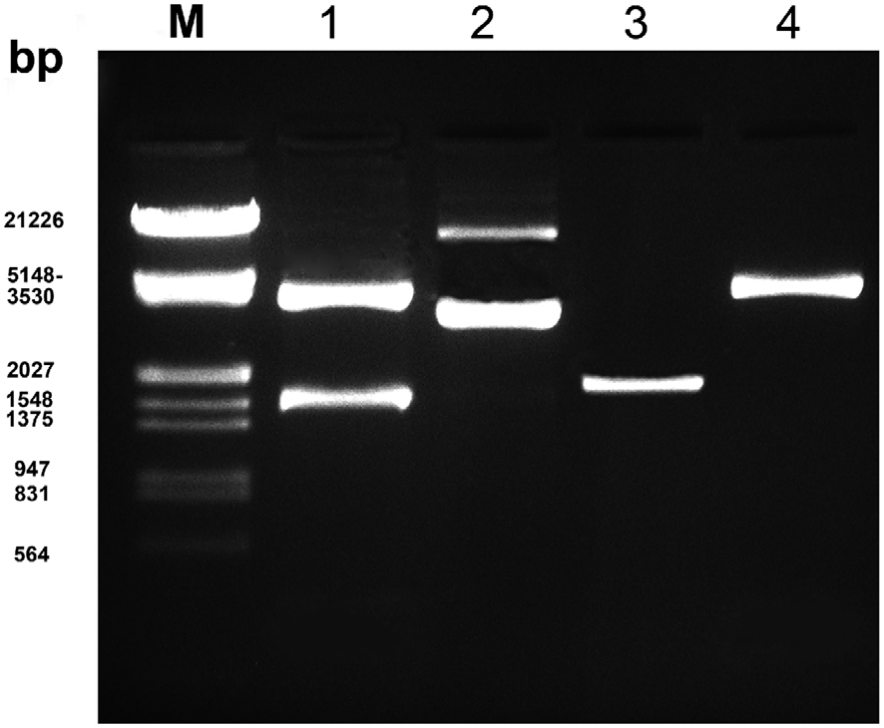
Electrophoretic analysis of restriction mapping. Lane 1 shows the pCR®2.1-TOPO® vector (3923 bp) above, which is cleaved by *EcoRI* (Invitrogen, Karlsruhe) and below the amplified 18S rRNA gene fragment (1724 bp) with the correct orientation (lane 2 with the incorrect orientation). The controls show the amplified PCR product (lane 3) and vector without insert (lane 4). M: marker λ-EcoRI +HindIII-Marker-Mix 3 (Fermentas, St. Leon-Rot) 1 µg.

All collected acanthocephalans (Table 1) were identified to species level by using morphological characters and existing keys [2], [7], [15]–[19], [28]–[30]. Of the resulting host-parasite combinations, *Filisoma rizalinum* and *Rhadinorhynchus lintoni* are new host and locality records. We have sequenced nearly the complete 18S rRNA gene, using cloning techniques to obtain strong sequencing signals for the entire gene (Figure 1). Identical sequences that represent different host or geographic isolates of a particular species were only included once in the phylogenetic analyses. They, however, provide molecular information on the host specificity and zoogeography of the studied acanthocephalan species. The SSU rDNA sequences were newly generated for 13 taxa and added to the published data set (GenBank). Analyses of this dataset (excluding sites containing gaps) of 40 taxa in Bayesian Inference had considerable similarity to the Maximum Likelihood tree. The SSU sequence length in the constructed alignment ranged from 1,649 (*Plagiorhynchus cylindraceus*) to 2,090 (*Polyacanthorhynchus caballeroi*) bp (Table 2). Nucleotide frequencies were 0.2544 (A), 0.1965 (C), 0.2657 (G) and 0.2834 (T). The proportion of invariable sites equaled 0.1605 and the distribution of gamma shape parameter (Gd) was 0.5669 (Table 3).

**Table 1:** Newly collected acanthocephalans.

Species	Host	Source
*Bolbosoma vasculosum*	*Lepturacanthus savala*	Java, Indonesia
*Pomphorhynchus laevis*	*Platichthys flesus*	Baltic Sea
*Pomphorhynchus laevis*	*Rutilus rutilus*	Lippe River, NRW, Germany
*Echinorhynchus gadi*	*Gadus morhua*	Baltic Sea
*Echinorhynchus gadi*	*Macrourus berglax*	Irminger Sea, Greenland
*Echinorhynchus gadi*	*Platichthys flesus*	Baltic Sea
*Filisoma rizalinum*	*Scatophagus argus*	Java, Indonesia
*Rhadinorhynchus prists*	*Gempylus serpens*	Java, Indonesia
*Rhadinorhynchus lintoni*	*Selar crumenophthalmus*	Oahu, Hawaii
*Serrasentis sagittifer*	*Johnius coitor*	Java, Indonesia
*Southwellina hispida*	*Epinephelus coioides*	Java, Indonesia

Some species with identical sequence data have been collected from different hosts.

**Table 2:** Acanthocephala and Rotifera specimen information and GenBank accession numbers.

Species	Family	Host	18S-rDNA	Length bp	Aligned
*Acanthocephaloides propinquus*	Arythmacanthidae	*Gobius bucchichii*	AY830149	1727	1657
*Acanthocephalus dirus*	Echinorhynchidae	*Asselus aquaticus*	AY830151	1724	1654
*Acanthocephalus lucii*	Echinorhynchidae	*Perca fluviatilis*	AY830152	1725	1655
*Arythmorhynchus brevis*	Polymorphidae	*Nycticorax nycticorax*	AF064812	1784	1694
*Bolbosoma vasculosum*	Polymorphidae	*Lepturacanthus savala*	**this study**	1739	1653
*Corynosoma enhydri*	Polymorphidae	*Enhydra lutris*	AF001837	1747	1651
*Corynosoma magdaleni*	Polymorphidae	*Phoca hispida botnica*	EU267803	1722	1653
*Echinorhynchus gadi*	Echinorhynchidae	*Macrourus berglax*	**this study**	1745	1659
*Echinorhynchus truttae*	Echinorhynchidae	*Thymallus thymallus*	AY830156	1729	1659
*Filisoma bucerium*	Cavisomidae	*Kyphosus elegans*	AF064814	1744	1655
*Filisoma rizalinum*	Neoechinorhynchidae	*Scatophagus argus*	**this study**	1741	1652
*Floridosentis mugilis*	Neoechinorhynchidae	*Mugil cephalus*	AF064811	1760	1668
*Gorgorhynchoides bullocki*	Rhadinorhynchidae	*Eugerres plumieri*	AY830154	1720	1651
*Koronacantha mexicana*	Illiosentidae	*Pomadasys leuciscus*	AY830157	1688	1665
*Koronacantha pectinaria*	Illiosentidae	*Microlepidotus brevipinnis*	AF092433	1761	1673
*Leptorhynchoides thecatus*	Rhadinorhynchidae	*Lepomis cyanallus*	AF001840	1758	1663
*Macracanthorhynchus ingens*	Oligacanthorhynchidae	*Procyon lotor*	AF001844	1765	1669
*Moniliformis moniliformis*	Moniliformidae	*Rattus rattus*	Z19562	1769	1668
*Neoechinorhynchus crassus*	Neoechinorhynchidae	*Catostomus commersoni*	AF001842	1773	1677
*Neoechinorhynchus saginata*	Neoechinorhynchidae	not applicable	AY830150	1745	1675
*Oligacanthorhynchus tortuosa*	Oligacanthorhynchidae	*Didelphis virginiana*	AF064817	1767	1671
*Plagiorhynchus cylindraceus*	Plagiorhynchidae	*Armadillidium vulgare*	AF001839	1745	1649
*Polyacanthorhynchus caballeroi*	Polyacanthorhynchidae	*Caiman yacare*	AF388660	2176	2090
*Polymorphus altmani*	Polymorphidae	*Enhydra lutris*	AF001838	1745	1649
*Polymorphus minutus*	Polymorphidae	*Gammarus pulex*	EU267806	1720	1651
*Pomphorhynchus laevis*	Pomphorhynchidae	*Rutilus rutilus*	**this study**	1742	1656
*Pomphorhynchus tereticollis*	Pomphorhynchidae	*Gammarus pulex*	AY423347	1662	1656
*Pseudocorynosoma anatrium*	Polymorphidae	*Bucephala albeola*	EU267801	1723	1654
*Pseudocorynosoma constrictum*	Polymorphidae	*Anas clypeata*	EU267800	1723	1654
*Pseudoleptorhynchoides lamothei*	Rhadinorhynchidae	*Ariopsis guatemalensis*	EU090950	1748	1663
*Rhadinorhynchus lintoni*	Rhadinorhynchidae	*Selar crumenophthalmus*	**this study**	1740	1653
*Rhadinorhynchus pristis*	Rhadinorhynchidae	*Gempylus serpens*	**this study**	1744	1656
*Serrasentis sagittifer*	Rhadinorhynchidae	*Platycephalus arenarius*	**this study**	1741	1654
*Southwellina hispida*	Polymorphidae	*Tigrisoma mexicanum*	EU267807	1730	1661
*Southwellina hispida*	Polymorphidae	*Epinephelus coioides*	**this study**	1747	1661
*Transvena annulospinosa*	Transvenidae	*Anampses neoguinaicus*	AY830153	1693	1656
Rotifera					
*Asplanchna sieboldi*	Asplanchnidae	Free-living	AF092434	1728	1663
*Brachionus patulus*	Branchionidae	Free-living	AF154568	1745	1656
*Lecane bulla*	Lecanidae	Free-living	AF154566	1733	1668

**Table 3:** Tree statistics for rDNA data set.

	Total characters	Uninformative-characters	Constant characters	Informative characters	CI	Tree length	-ln likelihood	Pinv	Gd
ML	2191	259	1224	708	0.547	2.866	16191.7480	0.1605	0.5669

Numbers of informative characters, consistency index (CI) and tree length refer to parsimony inference. Proportion of invariable sites (Pinv), shape of gamma distribution (Gd) and –ln Likelihood refer to Maximum Likelihood Inference.

### Phylogenetic analyses

**Figure 2:**
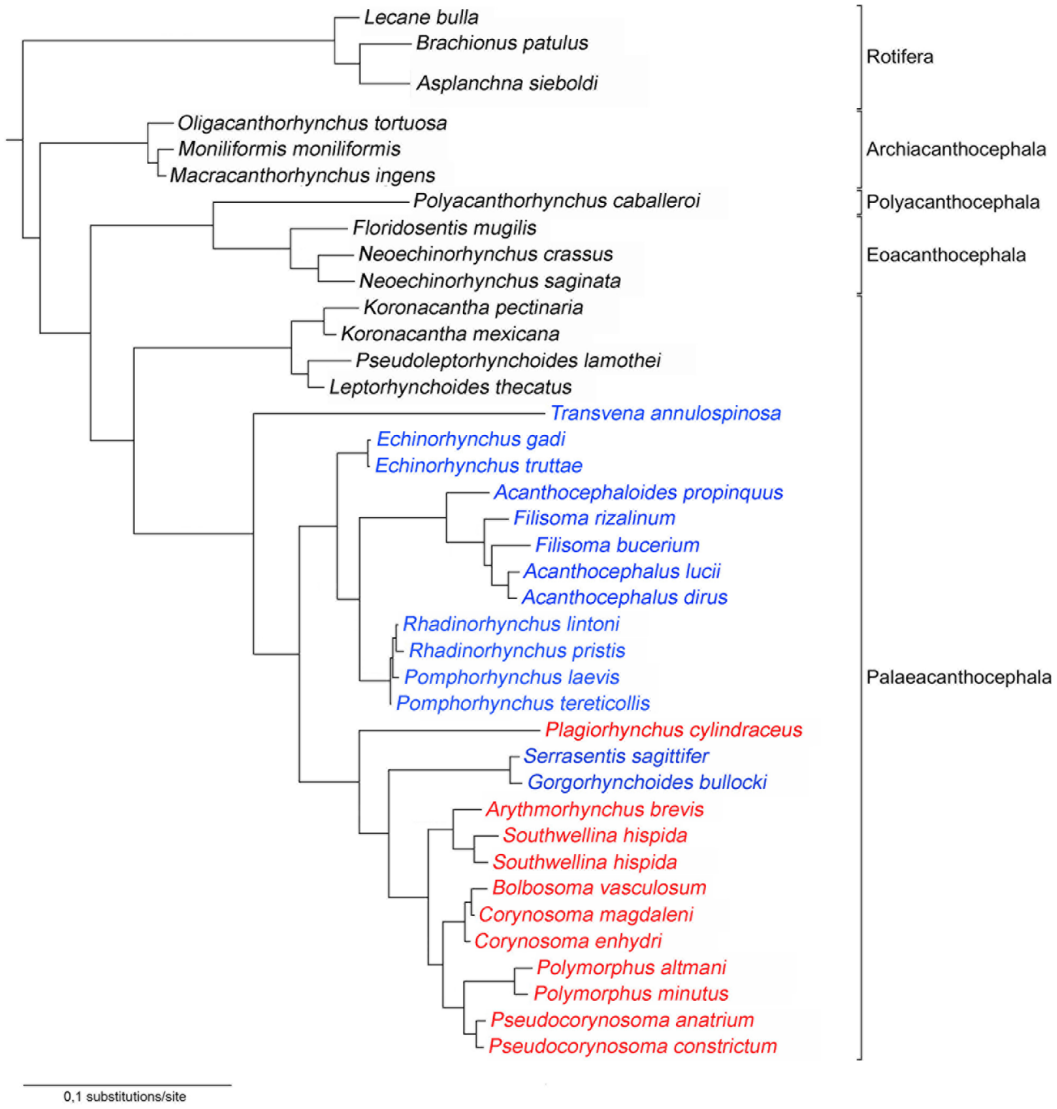
Bayesian consensus phylogram for Acanthocephala relationship based on the SSU rDNA data set. Rotifera is used as outgroup, acanthocephalans are classified as indicated on the right site of the graphic. This tree illustrates the hypothesis that the order Echinorhynchida (blue) and Polymorphida (red) have a paraphyletic arrangement. The branch length scale is the number of substitutions per site.

Bayesian Inference analysis yielded a single tree (Figure 2) with the same general topology as the ML result. Using this model the respective clades received high support in the ML bootstrap analysis. The topology of the BI tree depicts paraphyly of the Palaeacanthocephala.

Maximum-Likelihood analysis yielded a single best tree with a likelihood score of 16191.7480, a consistency index (CI) of 0.547 and a length of 2,866 steps (Table 2). The -ln likelihood score for the first alternative topology was 16182.22431. Based on the results the Kishino-Hasegawa (KH) [18] and Shimodaira-Hasegawa (SH) [19] tests were implemented in PAUP* using full optimization and 100 bootstrap replicates. Bootstrap values (<50%) are given on equivalent branches of the ML tree. The phylogenetic tree of the phylum Acanthocephala (Figure 2) is subdivided into four classes and the Rotifera as outgroup. The tree begins with the Archiacanthocephala as the earliest divergent clade, followed by the Polyacanthocephala and the Eoacanthocephala as sistertaxa, and the Palaeacanthocephala as the most derived clade. The Palaeacanthocephala show the highest diversity inside the class, presenting the orders Echinorhynchida and Polymorphida in a paraphyletic arrangement. All analyses support the current hypothesis separating four classes [((Eoacanthocephala, Polyacanthocephala) Palaeacanthocephala) (Archiacanthocephala), (Rotifera)], by Maximum-Likelihood trees and Bayesian Inference.

**Figure 3:**
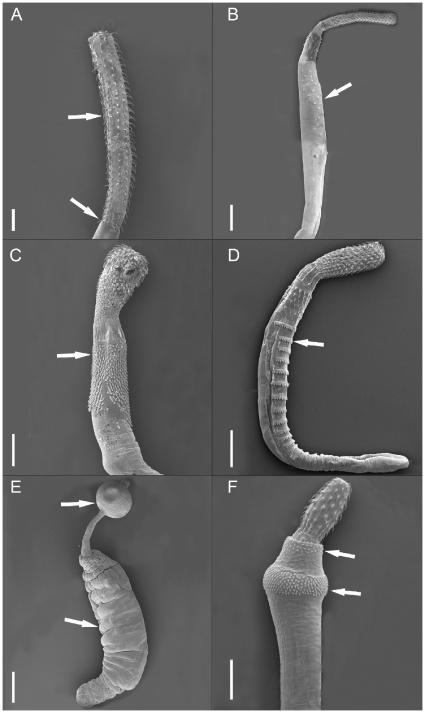
SEM (scanning electron microscope) micrographs of Palaeacanthocephala. (A) Proboscis of male *Rhadinorhynchus pristis* from *Gempylus serpens* (Indonesia, Indian Ocean) armed with regular hooks a and basal hook annulus. (B) Praesoma of female *R. lintoni* from *Selar crumenophthalmus* (Hawaii, Pacific) with irregular arrangement of trunk hooks. (C) Praesoma of *Gorgorhynchoides golvani* from *Platycephalus arenarius* (Indonesia, Indian Ocean) regular arrangement of surface hooks. (D) Habitus of *Serrasentis sagittifer* from *Platycephalus arenarius* (Indonesia, Indian Ocean) with hooks are transformed into strong plates arranged as combs. (E) Habitus of *Pomphorhynchus laevis* from *Platichthys flesus* (Baltic Sea) shows any trunk hooks on bulb, neck and trunk. (F) Praesoma of *Bolbosoma vasculosum* from *Lepturacanthus savala* (Indonesia, Indian Ocean) formed in the shape of a bulb, and armed with regular hooks which are arranged in two rings. Scale bars: A 400 µm, B, D, F, 200 µm, E 100 µm.

Defining morphological characters of the Archiacanthocephala are proboscis hooks in spirals, a single ligament sac in the females, and 8 cement glands in the males. The second clade consists of the Polyacanthocephala sister to the Eoacanthocephala. The Polyacanthocephala with the single genus Polyacanthorhynchus have 2 distinct ligament sacs in the females, and 2 elongate pyriform to tubular cement glands with giant nuclei in the males. The Eoacanthocephala with the representative *Neoechinorhynchus* are characterized by 2 ligament sacs in the females and a single cement gland in the males. The Palaeacanthocephala separate into the order Echinorhynchida as the original and the Polymorphida as the more derived taxon. The Echinorhynchida have an aspinosed trunk and a short neck. The cement glands of the males are divided into 2 or more compact or tubular lobes, and the females have eggs with polar prolongations of the middle shell. The final hosts are marine or aquatic fishes. The earliest divergent clade of the Echinorhynchida includes *Koronacantha*, *Pseudoleptorhynchoides* and *Leptorhynchoides*, which belong to the families *Illosentidae* and *Rhadinorhynchidae*. Koronacantha has an elongate proboscis with a heavy cuticular coating, cuticular body spines, genital spines are present in both sexes, the males have 8 cement glands, and the heavy, strongly recurved hooks in the shape of an inverted apostrophe with roots that are simple but exaggerated in size with a small hook. *Pseudoleptorhynchoides* and *Leptorhynchoides* have both, a cylindrical aspinose trunk, a cylindrical and elongated proboscis, and the males have 8 tubular cement glands. The next echinorhynchid taxon, *Transvena annulospinosa*, appears separate from the other 2 major clades. *Transvena* can be distinguished from all other Acanthocephala genera by having a combination of a single ring of small spines on its trunk near or at the junction between the neck and the trunk, and hooks which decrease in length from the apex to the base of the proboscis. The males have 2 pyriform or tubular cement glands. The next echinorhynchid clades lacks the 2 genera *Serrasentis* and *Gorgorhynchoides* (members of the echinorhynchids based on traditional classifications) (cp. Figures 3C,D), which appear in the polymorphid clade (Figure 2). *Echinorhynchus* is separated from the genera *Acanthocephaloides*, *Acanthocephalus*, and *Filisoma*, that form a sister group to *Rhadinorhynchus* and *Pomphorhynchus*. All these acanthocephalans are characterized by a slender cylindrical proboscis with many alternating longitudinal rows of homeomorphous hooks, the lack of surface hooks, and 4–6 cement glands in the males. The proboscis of *Rhadinorhynchus* shows a basal hook annulus, which is rudimentary in *Pomphorhynchus* and is absent in all other genera (Figures 3A,B,E). They are mainly fish parasites in the aquatic environment, including the common and widely distributed marine genus *Rhadinorhynchus*. The second clade of the Palaeacanthocephala consists of the *Polymorphida*, including the two echinorhynchid genera *Serrasentis* and *Gorgorhynchoides*. The most basal genus is the polymorphid *Plagiorhynchus cylindraceus* followed by a clade with the two echinorhynchids *Serrasentis sagittifer* and *Gorgorhynchoides bullocki*. The genus *Plagiorhynchus* can be distinguished from the remaining clade by the cylindrical fusiform aspinose trunk, slender lemnisci, and 6 elongate reniform or tubular cement glands in the males. The order Polymorphida is commonly characterized by the possession of alternating rows of hooks on the fusiform to globular proboscis, a mainly spinose trunk, surface hooks arranged in patterns, predominantly 2–4 cement glands in the males, and the final host specificity (adults in birds and mammals, juveniles in fishes, amphibians, and reptiles). The echinorhynchid genera *Serrasentis* and *Gorgorhynchoides* appear sister to the most derived monophyletic clade within the Palaeacanthocephala, within the polymorphids (Figure 2). According to morphology they demonstrate some polymorphid morphological characters, such as the spinose trunk and the rather globular, short calviform proboscis with longitudinal rows of variable numbers of hooks. While in *Gorgorhynchoides* the presence of trunk spines is limited to the anterior portion, *Serrasentis* has a trunk with unique ventral transverse rows of spines which are fused to form a comb-like structure (Figures 3C,D). The males have 6 clubbed cement glands (*G. bullocki*), and 4 elongate pyriform cement glands (*S. sagittifer*), which leads to the assignment into the Echinorhynchida based on morphology. Both genera occur mainly in fishes, rarely in amphibians, and in reptiles. The most derived genera within the present phylogenetic analyses belong to the Polymorphida, with the genera *Arhythmorhynchus* and *Southwellina* sister to *Polymorphus*, *Pseudocorynosoma*, *Bolbosoma*, and *Corynosoma*. While *Arhythmorhynchus* is characterized by an extremely long slender, anterior swollen trunk covered with a single field of spines, an usually enlarged cylindrical proboscis with greatly enlarged ventral hooks in the middle, and 2 (or 4) cement glands in the males, the genus *Southwellina* has a short trunk with spines that are arranged in 2 fields, and 4 tubular cement glands. Both parasitize birds as final hosts. *Bolbosoma* and *Corynosoma* are characterized by a small to medium sized body with a clubbed trunk, anteriorly swollen and armed with numerous regularly arranged spines. *Bolbosoma* is formed in the shape of a bulb, and is armed with spines that form 2 complete rings (see Figure 3F). The proboscis is calviform or conical, followed by a short neck, and the males have 2 tubular long cement glands. The trunk of *Corynosoma* is flattened on one side and forms a fore and a hind trunk. The spines are arranged within a single field, the proboscis is cylindrical, also followed by a short neck, and males have 6 pyriform or rarely tubular cement glands. Both genera use amphipods as intermediate, fishes as paratenic, and marine mammals as final hosts. *Polymorphus* and *Pseudocorynosoma* both show a spindle-shaped body armed with spines that are arranged in a single field, and a cylindrical or ovoid proboscis. *Polymorphus* has a small anterior spinose trunk, a cylindrical proboscis increasing in size proximally, a distinct neck region, and 4 tubular cement glands in the males. They prefer aquatic or semi aquatic birds, occasionally mammals, as final hosts. *Pseudocorynosoma* has a spindle-shaped body with a slight constriction, separating the fore and the hind trunk. Numerous spines that cover the most anterior part of the fore trunk are symmetrically distributed on the ventral and dorsal sides. In addition, a single field of spines is surrounding the genital pore. The proboscis has a slightly swollen region, followed by a truncated cone-shaped neck which is longer than wide [20], [22]. The males show 4 or 6 tubular cement glands. *Pseudocorynosoma* is using waterfowls as definitive hosts and amphipods as intermediate hosts.

## Discussion

The present study is the most detailed phylogenetic analyses of the Acanthocephala so far based on SSU rDNA, especially of the class Palaeacanthocephala. Earlier studies of acanthocephalans combining data sets of both, SSU and LSU (large subunit, already demonstrated similar results to the SSU alone [9]. Our data set adds to the most recent analyses of acanthocephalan relationships by Garey et al. [12] and García-Varela and Nadler [9]. We can support the notion that the acanthocephalans are monophyletic in origin, and separate into four distinct classes [2], [8], [9]. The Archiacanthocephala (Figure 2), parasites of birds and terrestrial vertebrates, are the earliest divergent lineage of acanthocephalans which utilize terrestrial vertebrates as intermediate hosts. More derived follow the Polyacanthocephalans as parasites of fishes and crocodiles, sister to the Eoacanthocephalans (in fish, amphibians and reptiles) from the aquatic environment. This result is consistent with the hypothesis that the Polyacanthocephala represent a different class within the phylum Acanthocephala. The more derived Palaeacanthocephala, including the Echinorhynchida and Polymorphida, are arranged in a paraphyletic assemblage. Both orders demonstrate high morphological diversity, which may explain why traditional identification keys have distinguished among the taxa according to their final hosts [1], [2]. The order Echinorhynchida infects teleost fishes, occasionally amphibians and reptiles whereas the Polymorphida include parasites of reptiles (rarely), birds, and marine mammals. The Echinorhynchida so far separate into 10 families and 339 valid species. The Polymorphida include only three families and a total of 255 valid species (Centrorhynchidae with two genera and 75 species; Plagiorhynchidae with 3 subfamilies and 8 genera and 53 species; Polymorphidae with 9 genera and 127 species). Consequently, these species rich taxa include 83 genera and 594 species of Acanthocephalans, mainly from the aquatic environment (Integrated Taxonomic Information System).

Herlyn et al. [14] for the first time described paraphyly within the Palaeacanthocephala, indicating independent evolution within these widely distributed taxa. Similarly, molecular and morphological studies so far indicated that the family Rhadinorhynchidae is paraphyletic or polyphyletic, and that the genera should be reexamined and reclassified by using morphological, ecological, and molecular characters [9], [21], [22], in agreement with the cladistic studies by García-Varela and Nadler [9] and Herlyn et al. [23]. The present analyses place the two species *Serrasentis sagittifer* (Rhadinorhynchidae) and *Gorgorhynchoides bullocki* (Rhadinorhynchidae), both Echinorhynchida, into the Polymorphida. Neither species demonstrates any morphological similarity. Conspicuous are the trunk hooks of *Serrasentis* that are arranged within rows (comb-like), and the presence of four cement glands in the males. *Gorgorhynchoides* has trunk hooks on its praesoma and six cement gland in the males (*Gorgorhynchoides golvani* from *Platycephalus arenarius*, Indonesia, Indian Ocean, see Figure 3). Most interesting is the position of the polymorphid *Plagoirhynchus cyndraecus*, which is arranged between the Echinorhynchida and Polymorphida. This species uses birds as final hosts. The cylindrical trunk also has anterior hooks around a small bulb, and the males have also six cement glands. According to traditional classifications, this result questions the relationship of *Serrasentis* and *Gorgorhynchoides* to the other echinorhynchids. While only some echinorhynchid acanthocephalans have mainly irregularly arranged surface hooks on the trunk, the herewith recognized character of regularly arranged hooks on the trunk is one of the most common features within the polymorphids.

Recent morphological assessment led to incongruent conclusions, due to difficulties in finding morphological characters that distinguish taxa, and to the partly subjective character states that often lack homologies with the outgroup [21]. According to García-Valera and Nadler [9], many families have been diagnosed based on character combinations rather than shared derived features. For several species, only a single record exists, caused by difficulties in sampling especially from the marine environment and in confirming the life cycles experimentally [1]. Most previous molecular approaches include too few acanthocephalan sequences, owed to difficult and/or biased sampling, to allow more detailed conclusions on the phyletic status of the acanthocephalan subclades [12], [14], [24], [25]. Nevertheless, with their relatively small number of species, a conserved two-host (arthropod–vertebrate) life cycle that involves paratenic hosts in the most derived clade, and the phylogenetic relationship to a free-living sister group, acanthocephalans are attractive candidates as model organisms for studying host-parasite co-evolution. For example, the species distribution within the host illustrates that fish and birds are the most widely used definitive hosts, followed by mammals. It is, however, interesting to note that the oldest group of vertebrates, the fish, is not utilized by significantly more species than the youngest groups, the birds and mammals [1], indicating expansive adaptive radiation in these newly explored host groups.

We are aware that the presented molecular phylogeny of the Acanthocephala is not yet comprehensive, and needs to be tested and validated by future studies. This requires further taxon sampling and ideally the inclusion of additional molecular markers. However, our data also demonstrate the preliminary nature of the acanthocephalan classification in general, especially of the derived echinorhynchids, the most common acanthocephalans in fish. We suggest that the current state of knowledge warrants the identification of further morphological characters for a better understanding of the acanthocephalan diversity, perhaps best driven by more in-depth molecular phylogenetic studies. This will enable the mapping of more morphological characters onto the molecular trees, and redefining the higher level classification of the Acanthocephala.

Acanthocephalans are attractive candidates as model organisms for studying the ecology and co-evolutionary history of parasitic life cycles in the marine ecosystem. However, the lack of phylogenetic studies and taxonomic identification of especially marine Acanthocephala prevents detailed comparison to other endoparasites. We do hope that our study will iniciate future research on the species composition, zoogeography and evolution of the phylum Acanthocephala, allowing comparisons to be made on the ecology of this taxon and other species groups such as the nematodes and cestodes that have diversified under similar conditions.

## Materials and Methods

### Ethics statement

An approval by a review board institution or ethics committee was not necessary, because all the fish in the current study were obtained in different locations from fishermen selling fresh fish for consumption or were collected during regularly fishery cruises.

### Collection of specimens

Acanthocephalan specimens were collected between 2001 and 2008 from their naturally infected vertebrate hosts (Table 1). The isolated parasites were washed in saline solution before fixation in 70% ethanol or absolute ethanol for molecular studies. The metasoma was used for molecular rDNA analyses, while the praesoma was processed for scanning electron microscopy (SEM). In other cases, the praesoma was stained in Mayer's acetic carmine, mounted in Canada balsam and identified using the common keys and original papers [26]–[28]. Molecular vouchers or voucher specimens were deposited in The Natural History Museum Berlin. A list of taxa, their place of origin and deposition numbers is given in Table 1.

### Nucleic acid isolation, polymerase chain reaction and sequencing

Genomic DNA was extracted from individual specimens using a commercial extraction kit (Peqlab, Erlangen). The region of nuclear rDNA was amplified using polymerase chain reaction (PCR). Nearly complete SSU rDNA (∼1.800 bp) regions were amplified after Garey et al. [12] (94°C 4-min initial denaturing followed by 30 cycles: 94°C 30 s, 60°C 30 s, 72°C 90 s) using primers corresponding to conserved regions at the extreme ends of the 18S rRNA gene (5′-AGATTAAGCCATGCATGCGTAAG-3′ and 5′-TGATCCTTCTGCAGGTTCACCTAC-3′), cloned into pCR®2.1-TOPO® vector (Invitrogen, Karlsruhe) and used to transform competent *Escherichia coli* (TOP 10, Invitrogen, Karlsruhe). Positive clones were identified by blue/white selection, and target inserts of white colonies were confirmed by PCR of bacterial DNA extracts. Liquid cultures for minipreps were grown in Luria broth containing 50 µg/µl of ampicillin following plasmid purification on the next day (MBI Fermentas, St. Leon-Rot). Orientation of cloned inserts was controlled by restriction mapping using in 1% agarose gel (Figure 1). Both strands of the 18S rDNA were sequenced completely in both directions after Sanger et al. [29] by Seqlab (Göttingen) using M13 universal primers (forward (−20): 5′ -GTAAAACGACGGCCAG-3′, reverse: 5′ -CAGGAAACAGCTATGAC-3′) of Invitrogen. Site polymorphisms were recorded only when both alternative nucleotide peaks were present in all sequence reactions representing both DNA strands. The sequences have been deposited in GenBank as given in Table 2.

### Alignment and phylogenetic analyses of sequence data

Sequences of the 18S rRNA gene of 11 sampled host-parasite combinations (Table 1) were aligned together with those from GenBank (Table 2), and included a total of 3 outgroup (Rotifera, belonging to the two major classes) and 36 ingroup (Acanthocephala) taxa (Table 2), representing the classes Archiacanthocephala (with three of four orders: Moniliformida, Gigantorhynchida and Oligacanthorhynchida), Eoacanthocephala (with one of two orders: Neoechinorhynchida) and Palaeacanthocephala (with two of two orders: Echinorhynchida and Polymorphida). The sequences were initially aligned using Clustal_X [30] and adjusted by eye. Based on these 40 sequences alignment had 2190 characters, 1902 were parsimony-informative. The complete alignment is available from the corresponding author upon request.

Phylogenetic trees were constructed using Bayesian Inference (BI) conducted with MrBayes v3.1.2 [31] and Maximum Likelihood (ML) with PAUP* v4.0b10 [32]. For BI, likelihood settings were set to nst = 6, rates = gamma, the nucleotide substitution model of evolution was the general time reversible (GTR) model [33], with invariable sites (+I) and rate heterogeneity (+G) [34] suggested as the best fitting model by Modeltest version 3.8 [35] based on Akaike Information Criterion (AIC). Four chains (one cold, three heated temp = 0.2) were run for 1,000,000 generations and sampled every 100 generations, whereas the 40,000 generations were discarded as ‘burnin’. For the calculated consensus tree a value of 0.95% and higher was considered having good statistical support.

For ML analyses, the same model parameters were used and heuristic searches were preset by nearest-neighbor-interchange (NNI), branch swapping was performed until the topology remained unchanged. Bootstrapping with 100 replicates was performed and the results were plotted onto the best known likelihood tree. Based on dataset BI analyses phylogenetic tree were reconstructed by TreeGraph [36] (Figure 2).
